# A dual transcriptional reporter and CDK-activity sensor marks cell cycle entry and progression in *C*. *elegans*

**DOI:** 10.1371/journal.pone.0171600

**Published:** 2017-02-03

**Authors:** Lotte M. van Rijnberk, Suzanne E. M. van der Horst, Sander van den Heuvel, Suzan Ruijtenberg

**Affiliations:** Developmental Biology, Department of Biology, Faculty of Science, Utrecht University Padualaan 8, CH Utrecht, The Netherlands; Institut de Genetique et Developpement de Rennes, FRANCE

## Abstract

Development, tissue homeostasis and tumor suppression depend critically on the correct regulation of cell division. Central in the cell division process is the decision whether to enter the next cell cycle and commit to going through the S and M phases, or to remain temporarily or permanently arrested. Cell cycle studies in genetic model systems could greatly benefit from visualizing cell cycle commitment in individual cells without the need of fixation. Here, we report the development and characterization of a reporter to monitor cell cycle entry in the nematode *C*. *elegans*. This reporter combines the *mcm-4* promoter, to reveal Rb/E2F-mediated transcriptional control, and a live-cell sensor for CDK-activity. The CDK sensor was recently developed for use in human cells and consists of a DNA Helicase fragment fused to eGFP. Upon phosphorylation by CDKs, this fusion protein changes in localization from the nucleus to the cytoplasm. The combined regulation of transcription and subcellular localization enabled us to visualize the moment of cell cycle entry in dividing seam cells during *C*. *elegans* larval development. This reporter is the first to reflect cell cycle commitment in *C*. *elegans* and will help further genetic studies of the mechanisms that underlie cell cycle entry and exit.

## Introduction

Cell division follows a sequence of events that together result in the segregation of replicated chromosomes and the formation of two new daughter cells. Creating cells in the correct numbers is critical to ensure proper development and tissue homeostasis, while imbalances between the formation and removal of cells within an organism can lead to cancer [1]. The most important decision to determine the creation of cells occurs in the G1 phase, when cells decide whether or not to enter a next cell division cycle. It has long been known that this decision depends on activation of cyclin-dependent kinases (CDKs) in association with G1 cyclins. External factors, such as the presence of nutrients (yeasts), growth factors and mitogens (multicellular organisms), determine G1 cyclin expression. In animals, growth factor signalling directly regulates the expression of D-type cyclins, while subsequent cyclin E transcription depends on activation of the cell division machinery. Cyclin D expression allows the formation of active CDK4/6-cyclin D complexes that phosphorylate the retinoblastoma protein (pRb). This reduces pRb-mediated inhibition of activating E2F transcription factors, and permits expression of E2F dependent cell cycle genes. Cyclin E is an E2F target, which upon expression can form an active kinase with CDK2 and further inactivates pRb. The pRb/E2F-cyclin E double-negative feedback loop creates a bistable switch, which likely governs commitment into the cell division cycle [2,3].

While cell cycle entry is not visible under the light microscope, discovery of the green fluorescent protein (GFP) as a biological marker made it possible to visualize activation of cell cycle genes by fluorescent protein expression [4]. Reporters containing E2F-dependent promoters, for instance of cyclin E and ribonucleotide reductase (*rnr*) subunit genes, have been used to visualize the moment cells come out of quiescence. Such reporters are less informative when examining continuously dividing cells or when determining cell cycle transitions. In addition to continued synthesis and GFP protein perdurance, such reporters do not reveal the balance between positive and negative regulators, which ultimately determines cell cycle entry [1]. For example, it has long been observed that contact inhibition and TGFβ exposure of mink lung epithelial cells leads to G1 arrest with substantial levels of CDK2 and cyclin E [5]. Activation of CDK2-cyclin E is prevented in these cells by expression of the CDK-inhibitory protein p27Kip1 [6]. Thus, while expression of G1 cyclins and E2F targets is a hallmark of cell cycle entry, this does not necessarily reflect the moment of cell cycle commitment.

Recently, improved fluorescent cell cycle reporters have been created that make use of cell cycle controlled degradation or localization of fluorescent fusion protein. This includes the fluorescent ubiquitination-based cell cycle indicator (FUCCI) reporter system, which visualizes the transition from G1 to S phase by a switch in the presence of red and green fluorescent proteins fused to the degradation signals of Geminin and Cdt1, respectively [7]. Moreover, a live-cell sensor that reflects CDK-activity rather than its presence was specifically designed to mark the moment of cell cycle commitment in human cells [8–10]. The CDK sensor consists of a DNA Helicase fragment linked to mVenus, which upon phosphorylation by active CDK2-cyclin E or CDK2-cyclin A relocates from the nucleus to the cytoplasm [8,9]. Expressing such a sensor within a genetic model system might allow detailed studies of cell cycle commitment in the context of development.

A model organism ideally suited for cell cycle studies with single cell resolution is the nematode *Caenorhabditis elegans* [11]. During *C*. *elegans* development, cells follow a stereotypic division pattern, with a strictly controlled and highly reproducible timing and frequency of cell division. *C*. *elegans* uses an evolutionarily conserved Rb pathway for cell cycle regulation, with expression of the G1 cyclins CYD-1^cyclin D^ and CYE-1^cyclin E^ corresponding to cell cycle entry [12,13]. The CDK-4^CDK4/6^–CYD-1 kinase is required for G1/S progression during post-embryonic development, and appears to counteract G1 inhibition by the pRb family member LIN-35 and APC/C coactivator FZR-1^Cdh1^ [12,14,15]. Live-observation of cell cycle transitions could greatly help cell cycle studies in *C*. *elegans*. The currently available tools for cell cycle analysis largely depend on fixation, e.g. on BrdU or EdU labelled larvae [11]. For developmentally-arrested cells, cell cycle entry can be visualized by induced expression of fluorescent proteins under the control of E2F target gene promoters (*rnr-1* and *mcm-4*) [13,14,16,17]. However, these reporters tend to be continuously expressed in lineages with rapidly proliferating cells, and both daughter cells remain fluorescent even after asymmetric cell divisions that produce one arrested daughter cell [17]. These findings highlight the need for additional reporters that allow the live visualization of cell cycle commitment.

Here, we describe the combination of the previously used *C*. *elegans* transcriptional reporter *mcm-4* with the live-cell CDK sensor as described in human cells for *in vivo* analysis of cell cycle entry during development in *C*. *elegans*. We show that this marker accurately reflects cell cycle entry and quiescence in divisions of the *C*. *elegans* seam cell lineage. This marker is the first available tool in *C*. *elegans* to visualize cell cycle commitment and will help future genetic studies of cell cycle entry.

## Results and discussion

### Development of a CDK sensor for *C*. *elegans*

**Fig 1:**
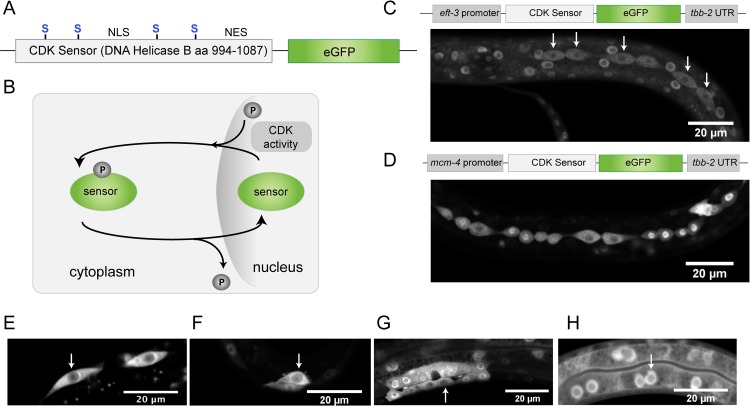
Dynamic localization of the CDK sensor in *C*. *elegans*. (A) The CDK-2 sensor consists of amino acids 994 till 1087 of Human DNA Helicase B with an NLS, NES, and four CDK target sites, and is fused to eGFP. Both the sensor and eGFP are codon optimized for use in *C*. *elegans*. (B) The phosphorylation status of the DHB fragment determines whether the sensor localizes to the nucleus or to the cytoplasm. As CDK-cyclin complexes are activated during cell cycle progression, the sensor becomes phosphorylated and accumulates in the cytoplasm. (C-H) Representative fluorescence microscopy images highlighting sensor expression and localization in a variety of tissues. (C) Expression of the sensor from the general *eft-3* promoter resulted in expression in all somatic cells of the animal. Arrow indicates seam cells in which the sensor is localized in the cytoplasm. (D-H) expression from the cell cycle regulated *mcm-4* promoter is specifically detected in cells that have the potential to divide, as shown for seam cells in an L3 larva (D). (E-H) Dynamic sensor localization was observed in seam cells (E), Q cells (F), vulval precursor cells (G), and to a lesser extent in intestinal cells (H).

In order to follow cell cycle progression in living *C*. *elegans* larvae, we aimed to create a fluorescent CDK sensor based on the previously described CDK reporter in human cells [9,18]. This sensor for CDK-activity consists of a part of the human DNA Helicase B fused to eGFP (DHB-eGFP), with a nuclear localization signal (NLS), nuclear export signal (NES), and four CDK target sites (Fig 1A). Phosphorylation is proposed to activate the NES and deactivate the NLS, resulting in cytoplasmic localization of the sensor (Fig 1B) [19]. Hence, high CDK-activity causes the sensor to localize to the cytoplasm. Cytoplasmic localization is therefore a measure of CDK-activity and cell cycle progression (Fig 1B).

We codon-optimized the DHB fragment for use in *C*. *elegans* and created a protein fusion with codon-optimized eGFP [20]. To drive expression, the sensor was placed under the control of the ubiquitously active *eft-3* promoter and *tbb-2* β-tubulin 3’ UTR. We used MosI-mediated single copy gene insertion (MosSCI) to generate transgenic lines with a single copy insertion of this CDK sensor transgene [21]. As expected, use of the *eft-3* promoter resulted in DHB-eGFP expression in all somatic cells of the animal (Fig 1C). While most cells in developing larvae showed nuclear fluorescence, distinct cells, as for example seam cells (Fig 1C, arrows) contained cytoplasmic localized DHB-eGFP, which corresponded to their expected time of proliferation. Thus, a live-cell CDK-activity reporter based on a human DNA Helicase B fusion protein appears suitable for use in *C*. *elegans*. At the same time, the universally high fluorescence levels made it difficult to detect the low numbers of cycling cells among the many quiescent and post-mitotic cells.

To restrict eGFP expression to proliferating cells, we decided to place the live-cell sensor under the control of a cell cycle-regulated promoter. In our previous studies, we observed high expression of MCM-4 (LIN-6) in all proliferating cells in *C*. *elegans* [17]. MCM-4 is one of the six subunits of the MCM2-7 replicative DNA helicase. The helicase activity is required for unwinding of the DNA in the licensing of DNA replication and during S phase. When expressed under the control of the *mcm-4* promoter, the sensor was only present in proliferating cells, in contrast to the *Peft-3*-based reporter (Fig 1C and 1D). Similar to the results obtained in human cells, a dynamic localization of the sensor was observed as cells progressed through the cell cycle. The translocation from the nucleus to the cytoplasm was clearly visible in cells of the epidermal seam and Q neuroblast lineages and vulva precursor cells (Fig 1E–1G). The sensor was also expressed in cells of the intestine, but nuclear localized fluorescence remained present in these cells, possibly reflecting their endo-replication cycles (Fig 1H). Overall, these observations indicate a wide dynamic range of sensor localization and its potential to visualize cell cycle progression.

### Visualization of cell cycle progression in the stem cell-like epidermal seam cells

**Fig 2:**
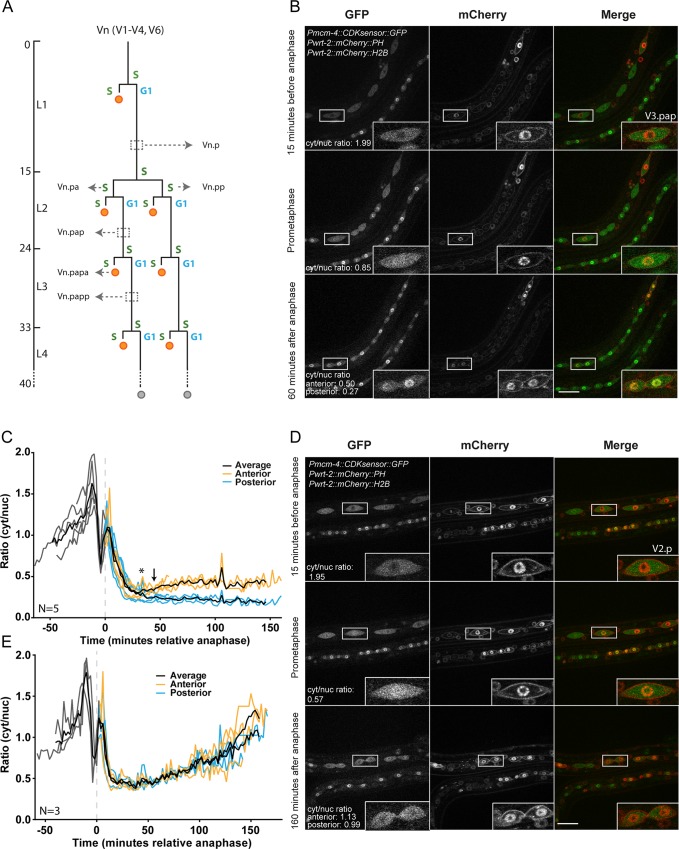
Sensor dynamics in L3 asymmetric and L2 symmetric seam cell divisions. (A) Schematic overview of divisions in the Vn seam cell lineages (n = 1–4, 6). These seam cells undergo one asymmetric cell division during every larval stage (L1-L4). During these divisions the seam cells produce a posterior self-renewing daughter cell (Vn.p), and an anterior daughter cell that differentiates and fuses with the hyp7 syncytium (Vn.a; orange circles). In the second larval stage (L2), seam cells undergo one additional, symmetric cell division, producing two self-renewing seam daughter cells. (B) Still images and blow-ups from spinning disk confocal fluorescence microscopy time-lapse movies of larvae staged at 24 hours after hatching. From top to bottom, images show seam cells (V3.pap, anterior to the left) before anaphase, during nuclear envelope breakdown at prometaphase, and after anaphase (V3.papa and V3.papp). Note that in the two newly formed daughter cells, nuclear export of the sensor is observed in the anterior daughter cell before it is observed in its posterior sister cell. Scale bar indicates 20 μm. (C) Graph representing the cytoplasmic-to-nuclear ratio (cyt/nuc) of DHB-eGFP in seam cell L3 asymmetric division (n = 5). Anterior cells in orange, posterior cells in blue, average ratio is indicated by a black line. Asterisk indicates the moment where plotted ratios in the anterior cell start to deviate from the ratios in the posterior cell. The arrow indicates the moment where the difference in ratios is statistically significant (t = 44 min after anaphase). Cells are aligned at the moment of anaphase (t = 0). (D) Still images and blow-ups of spinning disk confocal microscopy movies of worms staged at 20 hours after hatching. From top to bottom, images show seam cells (highlighted: V2.p, anterior to the left) before anaphase, during nuclear envelope breakdown at prometaphase, and after division (V2.pa and V2.pp). Note that in the two newly formed daughter cells the rate of nuclear export of the sensor is similar. Anterior to the left, scale bar indicates 20 μm. (E) Graph representing the cyt/nuc ratio of DHB-eGFP in symmetric L2 seam division (n = 3). Anterior cells in orange, posterior cells in blue, average ratio is indicated by a black line. The cells are aligned at the moment of anaphase (t = 0).

We further investigated the dynamics of CDK sensor localization for cells of the seam cell lineages, for which an interesting cell cycle regulation pattern has been described [22,23]. Seam cells form two rows of epithelial cells on the lateral sides of the animal. During each larval stage, the seam cells go through one round of asymmetric cell division, which creates a novel seam cell and a daughter cell that forms differentiated neurons or fuses with the general syncytium of the skin (hyp7). Interestingly, the differentiating anterior daughter cells go through S phase before fusing with hyp7 (Fig 2A) [23]. The posterior daughters become quiescent and only progress into S phase around the transition to the next larval stage [16,23]. In the second larval stage, however, the seam cell number increases through an extra symmetric cell division, which is followed by S phase entry and asymmetric cell division of both daughter cells. We previously observed expression of MCM-4::mCherry in both seam daughter cells, after symmetric as well as asymmetric cell division, illustrating that this reporter reflects proliferation potential but not cell cycle commitment [17].

In order to investigate whether we could visualize the difference in cell cycle progression between the anterior and posterior seam daughter cells, we performed time-lapse imaging on living animals expressing the *Pmcm-4*::*DHB-eGFP* reporter. Using spinning disk confocal fluorescence microscopy, we followed seam cells for several hours during asymmetric divisions in the third larval stage (L3) (Fig 2B, S1 Mov). This revealed that the CDK sensor becomes increasingly localized in the cytoplasm as cells progress through the cell cycle, with the highest cytoplasmic levels observed just before mitosis and cytokinesis. In contrast, the sensor showed a strong nuclear localization in both newly formed daughter cells (Fig 2B). Interestingly, differences in sensor localization appeared over time between the anterior and posterior daughter cells (Fig 2B bottom, S1 Fig, S1 Mov). The differentiating (anterior) daughter cells that proceed through S phase show gradually reduced nuclear levels of the sensor and translocation to the cytoplasm, indicating activation of CDKs and entry into the next cell cycle. The sensor remained nuclear in the posterior cells at this time point (Fig 2B, bottom, S1 Fig, S1 Mov). These data demonstrate that combining the *mcm-4* promoter and CDK sensor creates a valuable marker for visualizing cell cycle progression, and allows determining cell-to-cell differences in the timing and speed of cell cycle entry.

In order to more precisely quantify sensor localization, we used ImageJ software to measure cytoplasmic and nuclear fluorescence intensities. Markers for the DNA (*Pwrt-2*::*H2B*::*mCherry*) and cell membrane (*Pwrt-2*::*PH*::*mCherry*) helped us to select regions of interest and regions for background subtraction (see Material and Methods for Extended experimental procedures). A cytoplasmic-to-nuclear ratio was calculated from these measurements to determine sensor dynamics, with a high ratio as a read-out for cytoplasmic localization and a low ratio indicating nuclear localization of the sensor (Fig 2C). Confirming the visual examinations mentioned above, the calculated ratios show an increase in the cytoplasmic-to-nuclear ratio as cells progressed through the cell cycle. At the time of prometaphase, this ratio suddenly dropped. We measured an apparent transient increase in ratio just after anaphase onset, although there is no nuclear membrane at this point and thus the nucleus cannot be defined. As soon as nuclei reformed, the cytoplasmic over nuclear ratio dropped from approximately 1 to 0.25 in both daughter cells. However, from approximately 30 minutes after anaphase onset, differences in sensor localization between the anterior and posterior cells could be observed (Fig 2C asterisk, S1 Mov). In the anterior daughter cell, the ratio started to climb, while a further decline was observed in the posterior cell (Fig 2C. S1 Mov). Approximately 45 minutes after anaphase onset, the difference between the anterior and posterior cell is significantly different (arrow in Fig 2C; p < 0.05 at 44 min. after anaphase). Because quantification is laborious and low numbers were examined, the fact that the difference in levels of nuclear GFP are detectable by visual inspection is particularly meaningful (S1 Fig, S1 Mov).The cytoplasmic-to-nuclear ratio of the anterior daughter cell increased to around 0.5, approximately two times higher than that of the posterior daughter cell, consistent with the fact that the anterior cell proceeds into S phase while the posterior cell remains in G1 (Fig 2A). These quantifications indicate the timing of S phase commitment in the anterior cells, as this likely coincides with or precedes the moment that the plotted ratios of this cell start to differ from those of the posterior cell (Fig 2C, asterisks and arrow). The same conclusion can be reached more easily by visual inspection of sensor localization, which provides a convenient method for detecting cell cycle entry (S1 Mov).

As mentioned above, seam cells go through a symmetric cell division in the second larval stage (L2). We analyzed the sensor dynamics in this division to further test the correlation between cytoplasmic localization and cell cycle entry (Fig 2D, S2 Mov). Before anaphase, cells going through a symmetric L2 division show localization dynamics that resemble those of cells initiating an asymmetric L3 division; there is a rise in the cytoplasmic over nuclear ratio prior to anaphase, followed by a rapid decrease. However, the localization dynamics after anaphase differed from those in L3 divisions. The ratio did not drop below approximately 0.35 and started to go up approximately 30–50 minutes after anaphase. Importantly, both daughter cells showed a very similar increase in cytoplasmic localization of the CDK sensor and these levels further increased to a similar ratio as observed prior to the preceding anaphase (Fig 2E, t = 160). This is in agreement with these cells progressing into the next division (asymmetric L2 division) immediately after the symmetric L2 division. Thus, the localization of the CDK sensor varies with cell cycle commitment and cell cycle progression, while previous reporters showed similar expression in the daughter cells of symmetric and asymmetric cell divisions [17]. Thus, the *Pmcm-4*::*DHB-eGFP* reporter appears suitable for monitoring cell cycle entry in *C*. *elegans*, which is difficult to visualize otherwise.

### The CDK sensor as a marker for S phase entry

The quantifications above show that the cytoplasmic-to-nuclear DHB-eGFP ratio drops strongly in mitosis, and continues to decline further in the G1 arrested posterior daughter cell of an asymmetric seam cell division. The anterior cell that re-enters S phase shows a gradual increase in cytoplasmic-to-nuclear DHB-eGFP, starting approximately 30–40 minutes after anaphase. The CDK sensor remained partly nuclear in this latter daughter cell, and DNA synthesis is expected to take place while the DHB-eGFP cytoplasmic-to-nuclear ratio remains below 0.5 (Fig 2C). We wondered whether such a low cyt/nuc ratio indeed corresponds to S phase, or whether it remains particularly low in the hyp7-destined daughter cells in which S phase is not followed by mitosis. To address this question, we set out to determine the sensor distribution during S phase of the mitotic seam cell cycles in the first larval stage (L1).

**Fig 3:**
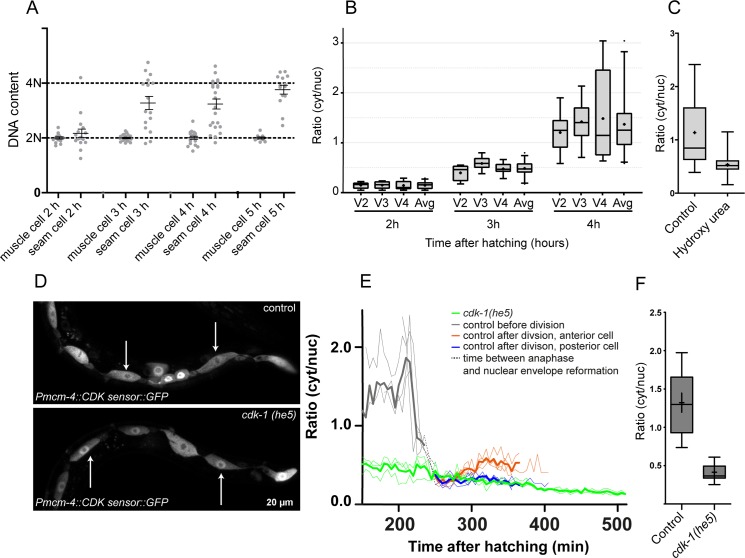
Sensor localization during S phase and CDK-1-dependent phosphorylation of the sensor. (A) Quantification of DNA content by propidium iodide staining in L1 larvae at 2, 3, 4 and 5 hours after hatching. Muscle cells were used as the reference for a 2N DNA content. Error bars indicate SEM. (B) Boxplots of ratios calculated from confocal fluorescence microscopy images of larvae staged at 2, 3 and 4 hours after hatching (n = 9 for single seam cells, n = 27 for average of cells). Avg. refers to the average ratio of V2, V3 and V4 together. The borders of the boxes are the 25^th^ and 75^th^ percentile, • indicates the mean, error bars correspond to 1.5× the interquartile range, outliers are shown. (C) Boxplots containing the quantification of cytoplasmic-to-nuclear fluorescence ratio in control and HU-treated larvae, 5 hours after hatching (n = 9). The borders of the boxes are the 25^th^ and 75^th^ percentile, the mean is indicated by **+**, error bars correlate to 1.5× the interquartile range. (D) Live-cell imaging of WT (top) and *cdk-1(he5)* mutant (bottom) larvae expressing the CDK sensor, at 300 minutes (5 hours) after hatching. (E) Spinning disk confocal fluorescence microscopy time-lapse movie analysis of sensor localization in control animals (n = 3 cells) and *cdk-1(he5)* mutants in L1 (n = 3). Control cells before anaphase in grey, control anterior cells in orange, control posterior cells in blue, *cdk-1(he5)* mutant cells in green. Average ratio is indicated by a bold line, individual cells are shown with thin lines. Dotted black lines indicate the time between anaphase and nuclear envelope reformation, where ratios could not be determined because of absence of the nucleus. (F) Comparison between the maximal calculated ratio’s in control (n = 10) and *cdk-1(he5)* (n = 14) animals during L1 development. The borders of the boxes are the 25^th^ and 75^th^ percentile, the mean is indicated by–, error bars correlate to 1.5× the interquartile range.

To determine the timing of S phase in seam cells in L1, we made use of synchronously grown larvae that were fixed at distinct time points. Initially, we used growth on EdU containing bacteria to be able to detect new DNA synthesis. At 4 hours of larval development, incorporation of EdU could be detected in part of the seam cells, but this method did not seem sensitive enough to visualize the initiation of DNA synthesis (S2 Fig). As an alternative method, we used propidium iodide (PI) incorporation to determine the DNA content in larvae fixed at subsequent time points (Fig 3A) [11,14]. Quantification of PI staining confirmed that the timing of DNA synthesis varies between seam cells, and showed that most seam cells enter S phase between 2 and 3 hours of larval development (Fig 3A). This conclusion is in agreement with a previous report, which used expression of a *Prnr*::*tdTomato* reporter to estimate the timing of S phase in seam cells [24].

Next, we used live-cell imaging to examine the subcellular distribution of the CDK sensor around the timing of S phase in the L1 asymmetric seam cell divisions (Fig 3B). Consistent with the PI staining results, we observed a substantial rise in cytoplasmic localization of the CDK sensor between two and three hours of larval development. The distribution of the sensor varied somewhat between individual seam cells at 3 hours of larval development, but the average cytoplasmic-to-nuclear ratio was very close to 0.5 (0.49 ± 0.15) (Fig 3B). This corresponds to the ratio found in the anterior daughter cells going through S phase after asymmetric cell division (Fig 2C). Thus, activation of CDKs present in S phase (most likely CDK-2/CYE-1 and possibly CDK-2/CYA-1) results in partial translocation of the sensor to the cytoplasm, and a cytoplasmic-to-nuclear ratio of approximately 0.5. In contrast to the anterior cells after asymmetric divisions, however, the cytoplasmic accumulation continued to increase at later times of the mitotic L1 seam cell cycle. These data indicate that initial phosphorylation and nuclear export of the sensor during S phase is followed by increased phosphorylation during G2 and early mitosis (see below).

As an alternative and complementary method to determine the dynamics of the DHB-eGFP reporter in S phase, we exposed newly hatched larvae to hydroxyurea. Hydroxyurea blocks the ribonucleotide reductase enzyme and thereby abolishes the production of deoxyribonucleotides, causing cells to arrest in S phase (Fig 3C) [25,17]. Live-cell imaging revealed that in control animals at 5 hours after hatching, the sensor was mainly present in the cytoplasm of the seam cells. In contrast, in hydroxyurea treated animals, the sensor failed to fully exit the nucleus, resulting in a cytoplasmic-over-nuclear ratio of around 0.5, which differs significantly from control animals (p < 0.0001). Since hydroxyurea causes replication stress and contributes to activation of the intra-S phase checkpoint that inhibits CDKs [26], we cannot rule out the possibility that hydroxyurea itself may also have an inhibitory effect on CDK-activity and sensor localization. Nevertheless, these data are in agreement with the conclusion that partial translocation and a DHB-eGFP cytoplasmic-to-nuclear ratio of around 0.5 is indicative for seam cells going through S phase. In mitotic cell cycles, but not endoreplication cycles, a more complete relocalization of the sensor takes place during the late S and G2/M phases.

### Nuclear export of DHB-eGFP likely depends on sequential phosphorylation by CDK-2 and CDK-1

The DHB-mVenus live-cell sensor described in human cells has been suggested to be preferentially phosphorylated by CDK2-cyclin E and CDK2-cyclin A [9]. However, the increased cytoplasmic localization when cells get closer to mitosis appears to indicate a contribution of CDK1 in association with mitotic cyclins. CDK1 is the most potent CDK, which can phosphorylate overlapping targets and substitute for CDK2 in mouse development [27]. *C*. *elegans* CDK-1 is specifically required for mitosis: homozygous *cdk-1* mutants complete embryogenesis in the presence of maternal product, go through DNA replication, but fail to enter mitosis during larval development [28]. To examine the contribution of CDK-1 to the phosphorylation of the DHB-eGFP sensor, we made use of the *cdk-1(he5)* presumed null allele. Using live-imaging as described above, we analyzed sensor localization in the seam cells after hatching. Around 5 hours of larval development, wild-type seam cells showed predominantly cytoplasmic localization of the sensor prior to mitosis (Fig 3D, top). In *cdk-1* mutant larvae the sensor was only partially translocated to the cytoplasm and remained enriched in the nucleus (Fig 3D, bottom). We quantified the cytoplasmic-to-nuclear ratios starting at 2.5 hr of L1 development in *cdk-1* mutants and control animals. In control animals, sensor localization showed a very similar pattern as observed during the asymmetric L3 division: the cyt/nuc ratio increases towards mitoses, drops after anaphase, after which the ratio starts to rise in the anterior cell (Orange lines, Fig 3E) while remaining low in the posterior (Blue lines Fig 3E). In contrast to control animals, *cdk-1* mutants, showed a maximum ratio of around 0.5 (green lines Fig 3E), very similar to the ratio observed in the anterior daughter cells when they enter S-phase (30–40 min after anaphase), before fusing with the hyp-7 syncytium (Fig 2A, green and orange line 3E). Comparison between the maximum ratio observed in control and mutant animals showed that this difference in cyt/nuc ratio was statistically highly significant (p < 0.0001)(Fig 3F). Notably, the ratios gradually continued to decline in the *cdk-1* mutants as DHB-eGFP started to accumulate in the nucleus again (Fig 3E). This nuclear relocalization likely indicates the requirement for continued phosphorylation to promote nuclear export. Phosphatases probably dephosphorylate the sensor, leading to nuclear re-accumulation after S phase when CDK-1 kinase activity is absent. Taken together, these data show that CDK-1 contributes to phosphorylation and translocation of the DHB-eGFP CDK sensor in *C*. *elegans*. However, the initial phosphorylation seems to be mediated by other kinases, possibly by the S-phase kinase CDK-2 in association with CYE-1^cyclin E^ as described for human cells [9].

In summary, we have successfully created a *C*. *elegans* CDK sensor, making use of the previously reported live-cell sensor for human cells in culture. This sensor provides an attractive tool for monitoring cell cycle progression during development, with single cell resolution and in real time. Combining the CDK sensor with an *mcm-4* promoter restricted fluorescence to cells with proliferation potential. This greatly helped recognizing and characterizing the cells of interest. We already made use of the sensor to determine the approximate timing of cell-cycle commitment of seam cells after symmetric and asymmetric cell division. We observed that cytoplasmic localization of the sensor depends on continuous CDK phosphorylation, and requires CDK-1 in G2/M. We propose a model in which the sensor could initially be phosphorylated by CDK-2/CYE-1 cyclin E in late G1/early S phase, which reveals the moment of cell cycle commitment. Subsequent phosphorylation by other kinases, possibly CDK-2/CYA-1 cyclin A in late S, and CDK-1/CYA-1 and CDK-1/CYB-1-3 cyclin B in G2 and mitosis are likely to further promote nuclear export. Based on the observed pattern of sensor localization we conclude that the CDK-1 kinase remains inactive during the endoreplication cycles of seam and intestinal cells.

## Materials and methods

### *C*. *elegans* strains and culture

Animals were cultured on NGM plates seeded with OP50 bacteria according to standard protocol. All strains were maintained at either 15 or 20°C. N2 Bristol is used as the wild type. Strains used are listed in S1 Table. Hydroxyurea was supplied to worms by adding 0,5 mL 500 mM hydroxyurea to NGM plates (containing approximately 10 gr NGM) before seeding, which after diffusion results in a 25 mM concentration. Larvae were synchronized by hypochlorite treatment and hatching of L1 animals in M9 medium + 0.05% Tween-20. Alternatively, for spinning disk confocal fluorescence microscopy time-lapse movies, larvae were staged by sequential wash-off from plates with egg-laying adults.

### Molecular cloning

Primers are listed in S2 Table. *Peft-3*::CDK *sensor* construct was made by Gibson Assembly of fragments of the CDK *sensor* (made by PCR of a G block containing the sensor with primers 422 and 423), eGFP (made by PCR from a vector containing eGFP with primers 424 and 425) and *tbb-2 3’UTR* (made by PCR from a vector containing the *tbb-2 UTR* with primers 426 and 427) into a MosSCI-II vector containing the *eft-3* promoter (cut with AscI and SbfI). *Pmcm-4*::CDK *sensor* construct was made by ligating the *mcm-4* promoter (made by PCR from a vector containing *Pmcm-4* with primers 428 and 429 and digestion with SpeI and AcsI) into the previously described *Peft-3*::CDK sensor construct (digested with SpeI and AcsI).

### Microinjection and transformation

Microinjection was performed as described previously [11]. For MosSCI integration, mos II worms were injected with 30 ng/μL mos transposase, 10 ng/μL of MosSCI-II construct containing the desired insert and 2,5 ng/μL *myo-2*::*TdTomato* together with 10 ng/μL *sur-5*::*dsRed* as co-injection marker.

### Live cell imaging by confocal microscopy

Images of worms were obtained using a Zeiss LSM700 Confocal microscope, at a magnification of 40×. Worms were synchronized by hypochlorite treatment and hatching L1 animals in M9 medium + 0.05% Tween-20. Slide preparation was performed by washing off and spinning down animals, and transferring 2 μL of worm pellet to 4% agarose slides with 2 μL 10mM tetramisole.

### Live cell imaging by spinning disk confocal microscopy

Worms were synchronized by wash-off staging. For L2 and L3 worms, slide preparation was performed by washing off and spinning down animals, and transferring 3 μL of worm pellet to 5% agarose slides with 1 μL 10 mM muscimol. For L1 worms, 7% agarose slides were used and muscimol was replaced by 10 mM tetramisole (2 μL tetramisole with 2 μL worms). L2 and L3 worms were imaged with a Nikon Eclipse Ti-U, using the following Microscope settings: Laser 488: 8% 150 ms, Laser 563: 10% 150 ms, 2x2 binning, no averaging, every 2 minutes z-stack with 3 frames of 0.5 micron. Microscope settings for L1 worms are: Laser 488: 8% 150 ms, Laser 563: 10% 150 ms, 2x2 binning, no averaging, every 5 minutes z-stack with 6–8 frames of 0.1 micron.

### Edu and pi staining

Immunohistochemical analyses and staining of DNA with propidium iodide and DAPI were performed as previously described [11]. EdU labelling and staining were performed according to a protocol using the Click-IT EdU Alexa Fluor 594 kit (Life Technologies) as previously described [11]. Primary and secondary antibodies used for immunofluorescent staining are as follows: mouse anti-AJM-1 (1:20, MH27, Developmental Studies Hybridoma Bank), goat anti-mouse-Alexa488 and goat anti-mouse-Alexa568 (1:500 Developmental Studies Hybdridoma Bank).

### Analysis and quantification of sensor localization

Sensor localization was quantified by measuring the cytoplasmic-to-nuclear ratio (cyt/nuc ratio) using Fiji (ImageJ). Regions of interest, the nucleus and cytoplasm, were drawn either by hand (freehand selection tool) or using the ‘analyze particles’ function as described in supplemental Methods. Measurements were obtained from the image after background subtraction, and were used to calculate the cyt/nuc ratio by dividing cytoplasmic intensity by nuclear intensity.

## Supporting information

S1 Table *C*. *elegans* strains. (PDF)

S2 Table Primers and oligos. (PDF)

S1 Fig Still images and blow-ups from spinning disk confocal fluorescence microscopy time-lapse movies of larvae staged at 24 hours after hatching. Highlighted area and magnification show the anterior (left) and posterior (right) daughter cells of V3.pap, 46 min. after anaphase. Note that a lower nuclear signal of the sensor is present in the anterior daughter cell as compared to the posterior sister cell. Scale bar indicates 20 μm.
(TIF)

S2 Fig EdU incorporation in seam cells during larval development. Representative fluorescence microscopy images of EdU, AJM-1 and DAPI staining of fixed worms. Ventral is up in the worm of 2 hours. Error bars indicate 20 μm.
(TIF)

S1 Mov Spinning disk confocal fluorescence microscopy time-lapse movie of larvae staged at 24 hours after hatching. In the top animal, from top to bottom, movie shows seam cells (anterior to the left) before anaphase, during nuclear envelope breakdown at prometaphase, and after anaphase.
(MP4)

S2 Mov Spinning disk confocal fluorescence microscopy time-lapse movie of larvae staged at 20 hours after hatching. In the top animal, movie shows seam cells (anterior to the left) before anaphase, during nuclear envelope breakdown at prometaphase, and after anaphase.
(MP4)

S1 Appendix Supporting materials and methods. (PDF)
