# Supplementary material for: A dual transcriptional reporter and CDK-activity sensor marks cell cycle entry and progression in C. elegans
Source: PLoS One. 2017 Feb 3;12(2):e0171600. doi: 10.1371/journal.pone.0171600 (PMC5291519; doi:10.1371/journal.pone.0171600)
Supplement: S1 Table — (PDF) [file pone.0171600.s001.pdf]

Table S1: ***C. elegans*** **strains**

**Strain #**	**Description**	**Additional information**
KN2595	*dpy-20 (e1362) ; huIs166 [Pwrt-2::mCherry::PH Pwrt-2::mCherry::H2B dpy-20 (+)]*	
SV93	*ncc-1(he5)/qC1dpy-19(e1259) glp-1(q339)III*	
SV1667	*unc-119 (edIII) ; heSi191 [Peft-3::CDK sensor::eGFP::tbb-2 UTR + Cbr-unc119(+)] II, line 1*	
SV1668	*unc-119 (edIII) ; heSi192 [Peft-3::CDK sensor::eGFP::tbb-2 UTR + Cbr-unc119(+)] II, line 2*	
SV1669	*unc-119 (edIII) ; heSi193 [Pmcm-4::CDK sensor::eGFP::tbb-2 UTR + Cbr-unc119(+)] II*	
SV1691	*unc-119 (ed3) III ; heSi191 [Peft-3::CDK sensor::eGFP::tbb-2 UTR + Cbr-unc119(+)] II ; dpy-20 (e1362) ; [Pwrt-2::mCherry::PH Pwrt-2::mCherry::H2B dpy-20(+)]*	Some silencing of mCherry in seam cells
SV1692	*unc-119 (ed3) III ; heSi191 [Peft-3::CDK sensor::eGFP::tbb-2 UTR + Cbr-unc119(+)] II ; dpy-20 (e1362) ; [Pwrt-2::mCherry::PH Pwrt-2::mCherry::H2B dpy-20(+) lin-48::eGFP]*	Some silencing of mCherry in seam cells
SV1693	*unc-119 (ed3) III ; heSi191 [Peft-3::CDK sensor::eGFP::tbb-2 UTR + Cbr-unc119(+)] II ; [Peft-3::TdTomato::H2B::unc-54 3'UTR + cbb-unc-199(+)]*	
SV1694	*unc-119 (ed3) III ; heSi193 [Pmcm-4::CDK sensor::eGFP::tbb-2 UTR + Cbr-unc119(+)] II ; dpy-20 (e1362) ; [Pwrt-2::mCherry::PH Pwrt-2::mCherry::H2B dpy-20(+)]*	Some silencing of mCherry in seam cells
SV1807	*unc-119 (ed3) III ; heSi193 [Pmcm-4::CDK sensor::eGFP::tbb-2 UTR + Cbr-unc119(+)] II ; oxTi619 [Peft-3::TdTomato::H2B::unc-54 3'UTR + Cbr-unc119(+)] ; ncc-1(he5) III, made by crossing EG7898 × SV1669 × SV93*	
SV1808	*unc-119 (edIII) ; heSi193 [Pmcm-4::CDK sensor::eGFP::tbb-2 UTR + Cbr-unc119(+)] II ; oxTi619 [Peft-3::TdTomato::H2B::unc-54 3'UTR + Cbr-unc119(+)] II*	
