# Supplementary material for: A dual transcriptional reporter and CDK-activity sensor marks cell cycle entry and progression in C. elegans
Source: PLoS One. 2017 Feb 3;12(2):e0171600. doi: 10.1371/journal.pone.0171600 (PMC5291519; doi:10.1371/journal.pone.0171600)
Supplement: S2 Table — (PDF) [file pone.0171600.s002.pdf]

Table S2: Primers and oligos

#	Name	Sequence
422	*cdk-2 sensor F GA*	CTACCGTCCGCACTCTTCTTAC
423	*cdk-2 sensor R GA*	CAACAAGAATTGGGACAACTCCAGT
424	*GFP codopt F GA*	ATGAGTAAAGGAGAAGAACTTTTCACTGG
425	*GFP codopt R GA*	aagggaatgcttgaaaggattttgcatttatcgcggccgcTTATTTGTATAGTTCATCCATGCCATGTG
426	*tbb-2 F GA*	TTACACATGGCATGGATGAACTATACAAATAAgcggccgcgataaatgcaaaatcctttcaagcattcc
427	*tbb-2 R GA*	GTATCTAGAACCGGTGACGTCAC
428	*mcm-4p speI*	CTCACTAGTGATttagacatccacgtc
429	*mcm-4p acsI*	aaaggcgcgcctttctagctgcaaaaatttacagatttcgc
-	*G block CDK sensor*	CTCTCTACCGTCCGCACTCTTCTTACTTTTAAATTAAATTGTTTTTTTTTCAGTTGGGAAACACTTTGCTCAGGCGCGCCaaaaATGACCAACGACGTTACCTGGTCTGAGGCTTCCTCCCCAGACGAGCGTACCCTTACCTTCGCCGAGCGCTGGCAACTTTCCTCCCCAGACGGAGTCGATACCGACGACGACCTTCCAAAGTCTCGTGCTTCTAAGCGCACCTGCGGAGTTAACGACGACGAGTCTCCATCTAAGATCTTCATGGTTGGAGAGTCCCCACAAGTTTCCTCCCGTCTCCAAAACCTTCGTCTTAACAACCTTATCCCACGCCAACTCTTCAAGCCAACTGACAACCAAGAGACCgcggccgccATGAGTAAAGGAGAAGAACTTTTCACTGGAGTTGTCCCAATTCTTGTTG
