# Supplementary figures and images for: A dual transcriptional reporter and CDK-activity sensor marks cell cycle entry and progression in C. elegans
Source: PLoS One. 2017 Feb 3;12(2):e0171600. doi: 10.1371/journal.pone.0171600 (PMC5291519; doi:10.1371/journal.pone.0171600)

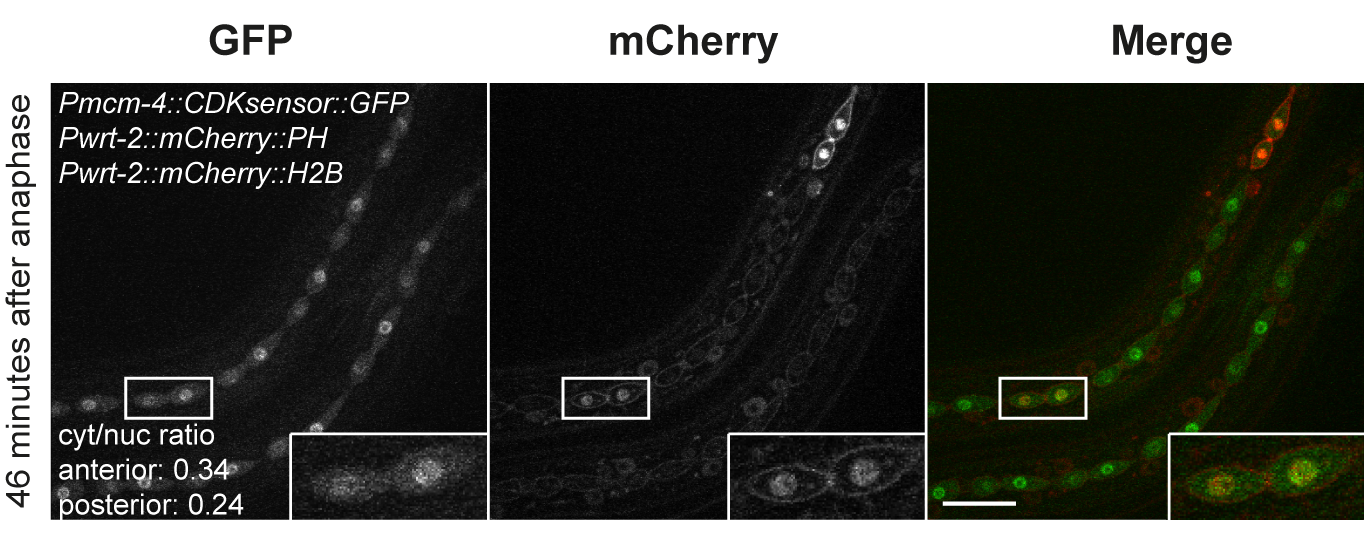

Supplement: S1 Fig — Highlighted area and magnification show the anterior (left) and posterior (right) daughter cells of V3.pap, 46 min. after anaphase. Note that a lower nuclear signal of the sensor is present in the anterior daughter cell as compared to the posterior sister cell. Scale bar indicates 20 μm. (TIF) [file pone.0171600.s003.tif]

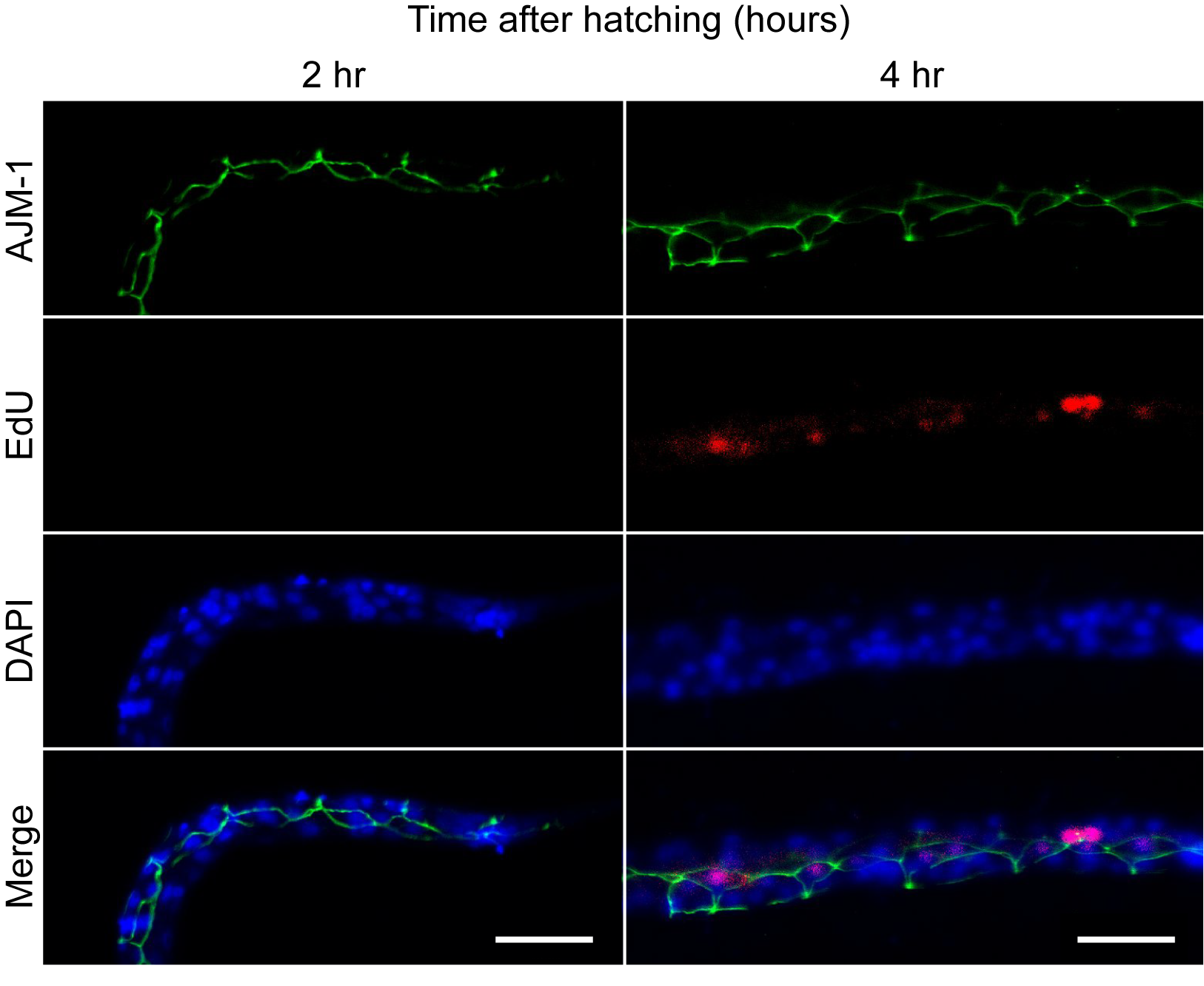

Supplement: S2 Fig — Representative fluorescence microscopy images of EdU, AJM-1 and DAPI staining of fixed worms. Ventral is up in the worm of 2 hours. Error bars indicate 20 μm. (TIF) [file pone.0171600.s004.tif]
