# Supplementary material for: A dual transcriptional reporter and CDK-activity sensor marks cell cycle entry and progression in C. elegans
Source: PLoS One. 2017 Feb 3;12(2):e0171600. doi: 10.1371/journal.pone.0171600 (PMC5291519; doi:10.1371/journal.pone.0171600)
Supplement: S1 Appendix — (PDF) [file pone.0171600.s007.pdf]

Supporting Material and Methods

Sensor localization was quantified by measuring the cytoplasmic to nuclear ratio using Fiji (ImageJ). Regions of interest, the nucleus and cytoplasm, were drawn either by hand (freehand selection tool) or using the ‘analyze particles’ function. For the latter method, first, the threshold was altered (at Image > Adjust > Threshold, note: check dark background) to a level where all desired regions are selected in red (either nuclei or entire cells). Second, the ‘analyze particles’ function was used (Analyze > Analyze Particles, note: adjust size and circularity to values only including the desired regions, i.e. size = 100-infinity, circularity = 0.2-1) to add all selected regions into the ROI manager. In this way, all nuclei outlines and outlines of the entire cells could be selected (either based on the sensor itself or on the nuclear and cell membrane markers). After retrieving the outlines of the nucleus and the cell outline, either drawn by hand or as described, a region of interest for the cytoplasm was created by selecting both ROIs from the ROI manager (Analyze > Tools > ROI manager), using the XOR function (in the ROI manager: more > XOR) and adding the newly created region to the ROI manager (ctrl+T). Measurements were performed after background subtraction. Background subtraction was performed by first duplicating the image or stack (ctrl+shift+D, note: check duplicate stack when analyzing a movie). On this duplicated file, the Gaussian blur filter was applied (Process > Filters > Gaussian Blur, note: sigma between 15 and 20, check preview to get the right amount of blurring, the outline of the worm should still be visible). Background was subtracted by the ‘image calculator’ function (Process > Image Calculator, note: set to subtract, and select the duplicated, blurred file to be subtracted from the original image). Measurements were then performed from the image after background subtraction, by selecting all ROIs from the ROI manager and selecting ‘measure’ (in the ROI manager menu).

These measurements were used to directly calculate the ratio of cytoplasmic intensity divided by nuclear intensity.
